# Tropane and Granatane Alkaloid Biosynthesis: A Systematic Analysis

**DOI:** 10.3390/molecules21111510

**Published:** 2016-11-11

**Authors:** Neill Kim, Olga Estrada, Benjamin Chavez, Charles Stewart, John C. D’Auria

**Affiliations:** 1 Department of Chemistry and Biochemistry, Texas Tech University, Lubbock, TX 79409-1061, USA; neill.kim@ttu.edu (N.K.); olga.peregrino@ttu.edu (O.E.); benjamin.chavez@ttu.edu (B.C.); 2 Office of Biotechnology, Iowa State University, Ames, IA 50011-1079, USA; cstewart@iastate.edu

**Keywords:** tropane alkaloids, granatane alkaloids, secondary metabolism, metabolic engineering

## Abstract

The tropane and granatane alkaloids belong to the larger pyrroline and piperidine classes of plant alkaloids, respectively. Their core structures share common moieties and their scattered distribution among angiosperms suggest that their biosynthesis may share common ancestry in some orders, while they may be independently derived in others. Tropane and granatane alkaloid diversity arises from the myriad modifications occurring to their core ring structures. Throughout much of human history, humans have cultivated tropane- and granatane-producing plants for their medicinal properties. This manuscript will discuss the diversity of their biological and ecological roles as well as what is known about the structural genes and enzymes responsible for their biosynthesis. In addition, modern approaches to producing some pharmaceutically important tropanes via metabolic engineering endeavors are discussed.

## 1. Introduction

Plants are sessile organisms and thus evolved natural products or “specialized metabolites” as a chemical response to both biotic and abiotic forces. Specialized metabolites are used by plants to defend themselves and communicate with other plants and organisms in their environments. Whilst the chemical diversity of plant specialized metabolites is vast, with total numbers thought to exceed over 200,000 structures, common themes of structure and function are the result of repeated and convergent evolution of both their biosynthesis and biological roles [1]. Moreover, the chemical structures and underlying biosynthetic enzymes of specialized metabolites serve as inspiration to medicinal and natural product chemists.

Many specialized metabolites are pharmacologically active and have been used by humans for therapeutic and recreational purposes since the beginning of recorded history. In particular alkaloids, pharmacologically active cyclic nitrogen containing metabolites derived from amino acids, are known for their pharmacological effects and frequently serve as the starting point for drug development [2]. Some of the oldest domesticated medicinal plants have been those that produce alkaloids. For example, *Erythroxylum coca*, a species notable for the production of the tropane alkaloid cocaine (**1**), was used in Peruvian households at least 8000 years ago [3]. Similarly, pomegranate (*Punica granatum*) has a history of cultivation that goes back at least 10,000 years in Egypt and is well-known for the production of granatane alkaloids [4].

**Figure 1:**
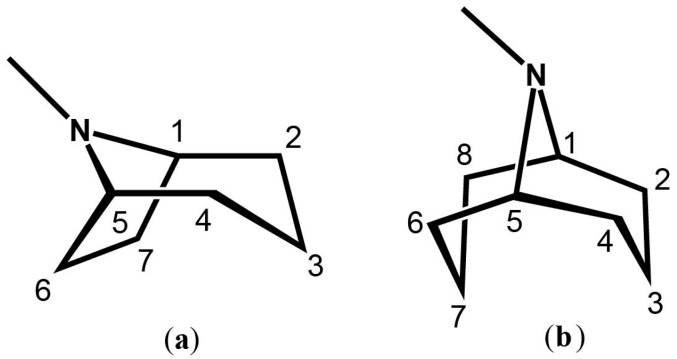
(**a**) The tropane core skeleton; (**b**) The bicyclic granatane core skeleton. Both structures depict their chemically accepted carbon numbering.

Tropane (TA) and granatane (GA) alkaloids are structural homologues, sharing similar chemical compositions and core scaffolds. Despite their similarities, TAs and GAs show different distribution patterns across the plant kingdom. The *N*-methyl-8-azabicyclo[3.2.1]-octane core structure of TAs is found in over 200 alkaloids [2,5,6] (Figure 1). In contrast, *N*-methyl-9-azabicyclo[3.3.1]-nonane, the core scaffold of GAs, appears in considerably fewer alkaloid metabolites (Figure 1). The bicyclic core structures of TAs and GAs differ by only one carbon atom, yet this difference alters the conformational preferences of each of the core skeletons [7]. Furthermore, the presence or absence of a single carbon atom in the core rings of TAs and GAs alters their chemical and pharmacological activities.

### 1.1. Similarities and Differences in Medicinal Properties

Despite their structural similarities TAs and GAs have distinct medicinal properties. TAs have been considered panaceas throughout recorded history, especially because of their anticholinergic properties. Anticholinergics are a class of compounds used as drugs to block the action of the acetylcholine neurotransmitter to treat motion sickness and diseases such as Alzheimer’s and Parkinson’s [8]. The methylated nitrogen in the core ring of cocaine (**1**) and other TAs serves as a structural analog of acetylcholine. TAs have been observed to attach to and inhibit muscarinic acetylcholine receptors [9]. TAs found in the Solanaceae are well known for both their anticholinergic and antispasmodic properties that affect the parasympathetic nervous system [10,11,12]. These plants have been used for pain relief, anesthesia, and as a treatment for drug addiction [10]. Daturae Flos, the dried flowers of *Datura metel* also known as “yangjinhua” in China, has been utilized and recorded in the Chinese Pharmacopoeia as an anesthetic and was prescribed to treat cough, asthma and convulsions [13]. *Przewalkia tangutica* is a rare medicinal solanaceous plant found in the Tibetan Plateau of China in which the roots, seeds and entire vegetative tissues are utilized [14]. *P. tangutica* contains several biologically active TAs including anisodamine (**2**), scopolamine (**3**) and atropine (the racemic mixture of hyoscyamine (**4**). The TAs present in *P. tangutica* are associated with many biological activities including analgesic, spasm modulation, pesticidal, and anti-inflammatory effects [14]. *Hyoscyamus niger*, also known as henbane, has been utilized in Chinese traditional therapy as well as in Tibetan medicine [15]. *H. niger* has been used as a sedative and sleep agent [16]. Hyoscyamine (**4**) and scopolamine (**3**) are the dominant TAs of *H. niger* and both metabolites can cross the blood-brain barrier to effect the central nervous system [17]. Scopolamine (**3**) has more potent pharmaceutical activity when compared to hyoscyamine (**4**) and exhibits relatively fewer side effects, however the scopolamine (**3**) content of solanaceous plants is usually much lower than the hyoscyamine (**4**) content [11]. Because of this, there is an ongoing effort to fully understand the biosynthesis of scopolamine (**3**) and other TAs within the Solanaceae (see Section 4).

The narcotic properties of cocaine (**1**), a TA from the non-solanaceous genus *Erythroxylum*, can be attributed to unique modifications of the TA core scaffold that are not present in TAs from solanaceous plants. The carboxylic acid methyl ester present at the C2 position is responsible for the binding of cocaine (**1**) to the dopamine transporter [18]. Cocaine (**1**) has also been reported to block the reuptake of nor-epinephrine, serotonin (5-HT receptor) and dopamine (D-A receptor) by the binding of the aromatic ring present at the 3β position of the molecule to specific sites in these receptors, affecting the normal physiology of the central nervous system [19]. This stereospecific conformation is dominant in TAs found in the Erythroxylaceae, but is only a minor constituent in solanaceous plants.

While no direct studies regarding the anticholinergic effects of GAs have been reported, a computational and NMR based study comparing the structures of TAs and GAs revealed that GAs adopt an *N*-axial form that is similar to many known TAs [20]. The *N*-methyl group in the axial conformation is thought to be the pharmacophore for TAs [21]. Additionally, the granatane ring system provides the semisynthetic intermediate for the potent antiemetic agents Dolasteron and Granisetron, which are serotonin 5-HT_3_ receptor agonists [22,23]. These compounds are used as medicines to prevent acute nausea in patients undergoing chemotherapy and radiotherapy for the treatment of cancer [24].

GAs and TAs have different physiological effects beyond the central nervous system. Unlike TAs, GAs exhibit anti-proliferative effects on hepatoma cells [24]. Specifically, murine hepatoma (BNL CL.2) and human hepatoma (HepG2) cell lines were cultured with various doses of crude GA-containing alkaloid fraction extracts from *Sedum sarmentosum*. Inhibition of excessive growth of tumor cells was observed, indicating that these compounds possess anti-cancer properties [25]. Other physiological effects that distinguish GAs from TAs include the use of GAs as an anti-worm treatment. Since the isolation of the first GAs, these compounds have been claimed to possess anti-worm (anthelminthic) capabilities, which were then studied in detail by scientists in the University of Amsterdam in 1956. These studies focused on deriving which GAs possessed the highest anthelminthic activity. The anthelminthic activity of synthetic granatane and its derivatives were measured in liver fluke. Their results rendered the highest anthelminthic activity to the compound isopelletierine [26]. These findings were later scientifically supported in 1963 with newer and better chemical methods [27]. Fascioliasis, a disease caused by liver fluke *Fasciola hepatica*, is common in cattle. Molluscidal activity in pomegranate bark extracts was effective in killing of *Lymnaea acuminata*, the vector for *F. hepatica* [28,29]. Beyond their medicinal properties, GAs also differ from TAs in that GAs have been found to be useful in the prevention of corrosion in the oil, gas and metal industries [30].

### 1.2. The Scattered Distribution of Tropanes and Granatanes amongst Angiosperms

Tropane alkaloids are commonly found in the genus *Erythroxylum* of the Erythroxylaceae family. The *Erythroxylum* genus includes at least 230 species that are distributed throughout the tropical regions of South and Central America [3,31,32]. *Erythroxylum coca* was one of the first domesticated plant species that provided nutritional, medicinal, and digestive properties to ancient civilizations by chewing the leaves of the plant [33]. Most of the cultivated coca used for cocaine (**1**) production comes from this species [34]. Albert Neimann first isolated cocaine (**1**) as a pure substance in 1860 [35] and its use exploded in popularity following an endorsement by Sigmund Freud [36]. The leaves of *Erythroxylum novogranatense* were also chewed by the elite class for their high content of methyl salicylate, which imparts a minty taste [37,38]. This species is known as “Colombian coca” and is found to be cultivated in the mountains of present day Colombia. “Trujillo coca” (*E. novogranatense* var. *truxillense*) is a cultivar that is grown in dry and arid regions. This species is also rich in methyl salicylate and contains other flavoring qualities that are still used in the production of Coca Cola, however today the extracts are decocainized [34].

Atropine, scopolamine (**3**), and hyoscyamine (**4**) are a few well-known TAs from the Solanaceae family. As discussed above, these compounds are commonly found in species such as *Atropa belladonna*, *H. niger*, and many members of the genus *Datura*. Scopolamine (**3**) was first isolated in 1888 from *Scopolia japonica* [39]. In medieval Europe, extracts from *A. belladonna*, were used as poisons, hallucinogens and aphrodisiacs [17]. Five to ten berries of *A. belladonna* could kill a person. The toxicity of the extracts has also been used on arrows to poison victims [40]. When extracts of *A. belladonna* are applied to the eyes, dilation of the pupils occurs [41]. For this reason, women used *A. belladonna* as a cosmetic drug during the Renaissance. Women of the 15th century who were devoted to witchcraft also exploited the psychoactive effects of *A. belladonna* [16]. Mucous membranes, such as those found in the walls of the oral cavity and the vulva, are readily susceptible to drug absorption. It is believed that the application of alkaloid-containing salves to the skin or vulva was achieved by the use of brooms. It gave users the feeling of being able to fly, feeding the folkloric associations of witches with brooms [17]. Atropine was first isolated in 1833 from *A. belladonna* [42,43]. The correct structure of atropine was obtained by Willstätter in 1889 after much deliberation and structural studies [17]. Leaves of *D. metel* from solanaceous plants were used as herbal cigarettes in the 19th and 20th centuries to treat patients with asthma or other respiratory conditions [17].

**Figure 2:**
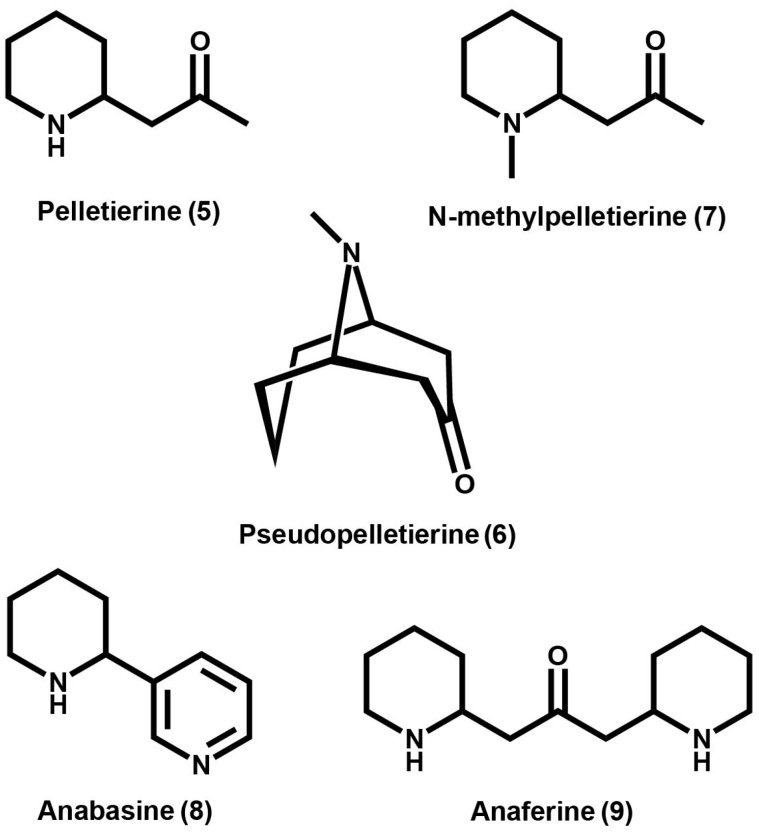
Examples of granatane alkaloids and granatane alkaloid derivatives found among several plant species.

GAs include pelletierine (**5**), isopelletierine, pseudopelletierine (**6**), and *N*-methylpelletierine (**7**) and their derivatives anabasine (**8**) and anaferine (**9**) (Figure 2). GAs are predominantly found in *P. granatum* although they have been characterized in other species such as *S. sarmentosum*, and *Withania somnifera* [44,45,46,47]. The pomegranate tree is native to Iran, Afghanistan, Baluchistan, and Himalayas in Northern India. Pomegranate can also be found in the Mediterranean and Caucasus regions due to its ancient cultivation. Today, pomegranate is cultivated all over India, Southeast Asia, Malaysia, the East Indies, tropical Africa, and the United States [48]. In 1879, French chemists Tanret and Pelletier isolated a basic substance from the root bark of the pomegranate tree and characterized the salt [45]. As of this review, no studies regarding characterization of any structural genes or the enzymes responsible for the production of GAs has been reported. Interestingly, the isolation of pseudopelletierine (**6**) has been reported in the species *Erythroxylum lucidum* [49] suggesting that GAs and TAs may use similar biosynthetic machinery.

**Figure 3:**
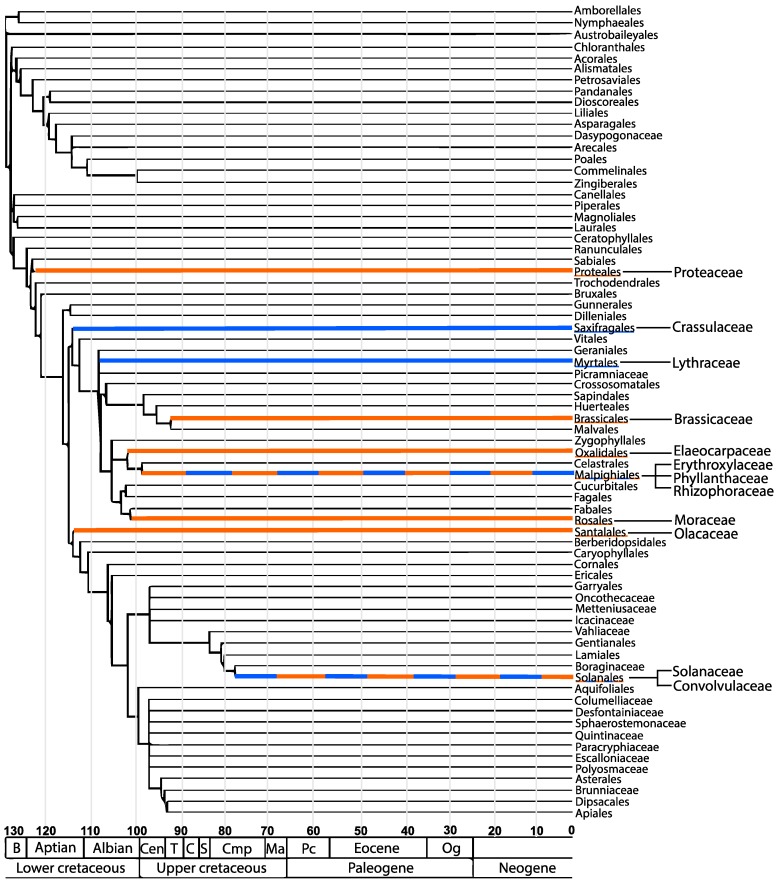
The diversity and similarities of tropane and granatane producing angiosperms. The orders tropane alkaloids belong to are highlighted in orange. The orders granatane alkaloids belong to are highlighted in blue. Shared orders are represented by both colors. The plant families that are known to produce tropane and granatane alkaloids are branched off from the orders. The scale at the bottom represents millions of years. Modification of the phylogenetic tree published in [54], republished with permission with the Botanical Society of America.

Scientific reports on the occurrence and distribution of TAs from angiosperm families other than the Solanaceae and Erythoxylaceae are few. When viewed against a phylogeny of angiosperms, the results reveal a scattered and non-contiguous distribution (Figure 3) [50,51]. For example, TAs have been reported in members of the family Proteaceae. Compounds found in the family Proteaceae include the pyranotropanes, strobamine and bellendine as well as the compounds ferruginine and ferugine [52]. In addition, a few members within the families Brassicaceae and Convolvulaceae have been found to produce calystegines, which are heavily hydroxylated forms of TAs [53]. The main TA producing species are scattered among four orders, which include the Solanales, Malpighiales, Proteales and Brassicales. In the case of GAs, *P. granatum* from the family Lythraceae is the main producing species. However, the appearance of GAs has also been reported in members of the Solanaceae, Crassulaceae, and Erythroxylaceae families. These families belong to the orders Myrtales, Solanales, Malpighiales, and Saxifragales. This distribution pattern raises the important question about the biosynthetic origin of the respective GA and TA pathway. Namely, have these biosynthetic pathways arisen from a common ancestor or have they arisen independently in several cases? Recent evidence in TA biosynthesis suggests that certain biosynthetic steps have multiple origins [5]. By extension, this could also mean that GA biosynthesis has arisen independently more than once. 

Examples of scattered distributions of alkaloid classes can be observed throughout the plant kingdom. For example, the legumes (Fabaceae) contain several alkaloid secondary metabolites that are not found in all members within the family [55]. In some cases, de novo evolution of the pathways have been observed. In the case of indolizidine alkaloids, the source of the scattered appearance of alkaloids is theorized to occur due to horizontal gene transfer from alkaloid producing endophytic fungi.

### 1.3. Biosynthesis of TAs and GAs

Diversity among tropane and granatane alkaloids can also be seen at the biochemical level. Most data available regarding enzymes involved in TA biosynthesis comes from species within the Solanaceae family. Despite recent advancements, some critical steps in TA biosynthesis remain ambiguous. Additionally, recent advances in high throughput sequencing technologies, plant genomics, and biochemical methods have aided the progress of elucidating TA biosynthesis in other non-model species, specifically within the Erythroxylaceae. Lastly, even though the biosynthesis of GAs is predicted to be similar to TAs, plausibly a whole new set of enzymes were recruited for the biosynthesis of GAs. This would mean that untapped gene and enzyme diversity is present in the GA and TA biosynthetic pathways. It is therefore important to elucidate the biosynthesis of these alkaloids to help further understand specific parts of their mechanisms and their metabolic processes.

## 2. Tropane Alkaloid Biosynthesis

**Scheme 1:**
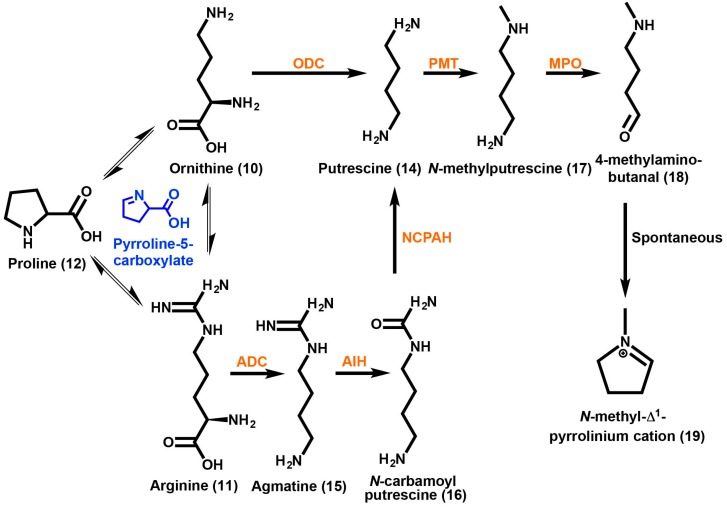
Tropane alkaloid biosynthesis up to the formation of the *N*-methyl-Δ^1^-pyrrolinium cation. Arginine, ornithine and proline are interconvertable, sharing the pyrroline-5-carboxylate intermediate (highlighted in blue). Putrescine can be formed directly via decarboxylation of ornithine or indirectly through arginine. Methylation of putrescine is followed by oxidation to yield 4-methylamino-butanal, which is then spontaneously cyclized to yield the *N*-methyl-Δ^1^-pyrrolinium cation. Enzymes are highlighted in orange and abbreviated as follows: ADC, arginine decarboxylase; AIH, agmatine imino hydrolase; NCPAH, *N*-carbamoylputrescine amino hydrolase; ODC, ornithine decarboxylase; PMT, putrescine *N*-methyl transferase; MPO, methyl putrescine oxidase.

Overall, the process of producing either TAs or GAs in plants begins with the diversion of specific amino acids from primary metabolism into the formation of an initial nitrogen-containing ringed compound. This heterocycle will in most cases proceed on to form a bicyclic structure in which the core skeletons are created (Figure 1). Further modifications will add diverse functional groups to the core structure yielding the final end product. Ornithine (**10**) and arginine (**11**) are the amino acids predicted to be the starting substrates of TA biosynthesis (Scheme 1) [56]. Feeding studies using ^14^C-proline into the roots of *A. belladonna* also suggest that proline (**12**) may be a starting amino acid for incorporation into the tropane moiety. Other studies using *D. metel* and *D. stramonium* have also shown the incorporation of proline (**12**) into TA compounds, such as scopolamine (**3**) and tropine (**13**) [57]. Biosynthetically each of these three amino acids can generate a pyrroline-5-carboxylate intermediate, which can be interconvertible between these amino acids [58]. Consequently, feeding studies of labeled amino acids are difficult to interpret without further enzymological data.

A nonsymmetrical intermediate has been proposed for the production of the pyrrolidine ring, if the amino acid ornithine (**10**) is first methylated at the γ-*N* position. A proposed alternative route includes the decarboxylation of ornithine (**10**) to form the polyamine putrescine (**14**) as the first step [59,60]. Feeding studies of radioactively labeled ornithine-2-^14^C have shown that several *Datura* species incorporate a nonsymmetrical intermediate [59]. However, a symmetrical intermediate has been reported for *Nicotiana*, *Erythroxylum*, and *Hyoscyamus* species [61]. Symmetrical incorporation showed activity at positions C1 and C5 of the tropane ring and has been reported for *Nicotiana*, *E. coca*, and *Hyoscyamus albus* [59,60]. A one-step enzymatic approach is possible to reach a symmetrical intermediate by converting ornithine (**10**) into putrescine (**14**) catalyzed by ornithine decarboxylase (ODC) [62]. Another route to putrescine (**14**) can be taken by starting with the amino acid arginine (**11**). The decarboxylation of arginine (**11**) into agmatine (**15**) is catalyzed by arginine decarboxylase (ADC). Agmatine (**15**) can then be converted into *N*-carbamoyl putrescine (**16**) via agmatine imino hydrolase (AIH). The enzyme *N*-carbamoyl putrescine amido hydrolase (NCPAH) then yields putrescine (**14**). Both the ADC and ODC directed pathways have different and diverse outcomes in regards to primary and secondary metabolism. Putrescine (**14**) that was produced by ODC is important for the supply of polyamines for primary metabolic processes such as cellular differentiation, development and division [63]. On the other hand, putrescine (**14**) that was produced by ADC in the Solanaceae is thought to be required for environmental stress related responses.

The formation of *N*-methylputrescine (**17**) catalyzed by putrescine *N*-methyltransferase (PMT) is considered the first rate-limiting step in the TA biosynthetic pathway [64]. PMT is an *S*-adenosylmethionine (SAM)-dependent methyltransferase that attaches a methyl group to the nitrogen atom that ultimately appears in the tropane skeleton. The first PMT sequence to be isolated from plants came from tobacco (*Nicotiana tabacum*) [65]. Since then, many other PMT related sequences have been isolated and characterized from other pyrrolidine alkaloid producing plant species. PMT belongs to a large family of enzymes that are involved in polyamine production. These primary metabolites play an important role in stress physiology, senescence, and morphogenesis [66]. Several lines of evidence suggest that PMT function has evolved from an ancestral spermidine or spermine synthases [67]. This would suggest that an enzyme from primary metabolism was recruited for the production of secondary metabolites. Localization of the PMT protein is root specific for both nicotine and tropane producing solanaceous plants [68,69,70]. While *N*-methylputrescine (**17**) is the predominant intermediate for solanaceous TAs, at least one alternative has been suggested. [6-^14^C]-1,5,10-triazadecane fed to *Nicotiana glutinosa* plants revealed that *N*-methylspermidine can also be incorporated into the pyrrolidine ring of nicotine [71].

The next step in TA biosynthesis is the formation of 4-methylamino-butanal (**18**) from *N*-methylputrescine (**17**) catalyzed by methylputrescine oxidase (MPO). This enzyme coexists with a class of copper-dependent diamine oxidases that uses copper as a cofactor to oxidize a conserved tyrosine residue into a topaquinone, essential for enzyme catalysis [72]. MPO was first characterized from the species *N. tabacum* [73,74]. Although an MPO like gene has yet to be discovered in *E. coca*, the corresponding activity from intact plants fed 4-monodeuterated *N*-methylputrescine shows the same enantioselectivity as solanaceous plants that produce either nicotine or TAs [75]. Using ^13^C fractionation techniques, researchers have found that both nicotine and hyoscyamine (**4**) share the same biosynthetic pathway, at least up to the *N*-methyl-Δ^1^-pyrrolinium cation (**19**) [76]. Subsequently, a hypothesis has been proposed that a metabolic channel prevails in which a multi-enzyme complex is active. The product of MPO, 4-methylamino-butanal (**18**), spontaneously cyclizes to form the *N*-methyl-Δ^1^-pyrrolinium cation (**19**) [59]. Feeding studies using ornithine-2-^14^C detected labeled 4-methylamino-butanal in *D. stramonium* plants [59]. The *N*-methyl-Δ^1^-pyrrolinium cation (**19**) serves as the first ring in the bicyclic tropane skeleton.

**Scheme 2:**
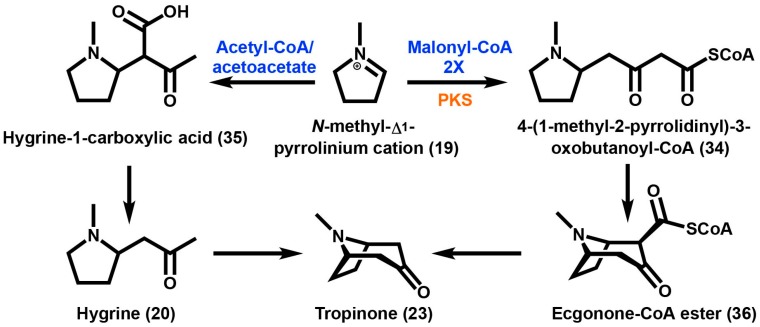
Tropinone formation from the *N*-methyl-Δ^1^-pyrrolinium cation in which there are two possibilities for the condensation of the tropane ring. Acetyl-CoA is utilized via acetoacetate to yield hygrine-1-carboxylic acid. On the other hand, two successive decarboxylative condensations of malonyl-CoA by PKS (polyketide synthase) yields 4-(1-methyl-2-pyrrolidinyl)-3-oxobutanoyl-CoA. Enzymes are highlighted in orange and substrates are highlighted in blue.

While the biochemical role of PMTs and MPOs are generally accepted steps leading to the first ring closure of tropane intermediates in the Solanaceae, the enzymatic basis of the second ring closure is controversial. For many years, the compound hygrine (**20**), (*R*)-1-(1-methylpyrrolidin-2-yl)-propan-2-one was thought to be a direct intermediate based on feeding studies, however more recent studies have demonstrated that previous results are likely experimental artifacts [77,78,79]. In solanaceous plants the best incorporation into the second ring was achieved from racemic ethyl [2,3-^13^C_2_]-4-(*N*-methyl-2-pyrrolidinyl)-3-oxobutanoate [77,80], a polyketide based molecule (Scheme 2). Further evidence for the involvement of a polyketide was demonstrated by the feeding of methyl (RS)-[1,2-^13^C_2_,1-^14^C]-4-(1-methyl-2-pyrrolidinyl)-3-oxobutanoate to the leaves of *E. coca* [81]. This strongly implicates the involvement of acetate-derived metabolites (e.g., polyketides) during the formation of the second ring in TAs. There are limited types of enzymes capable of catalyzing this type of condensation reaction. In the case of benzylisoquinoline alkaloid biosynthesis a Pictet–Spenglerase enzyme was shown to condense dopamine with 3,4-dihydroxyphenylacetaldehyde [82]. However, based on more recent isotope and radiolabeled feeding studies it is hypothesized that a type III polyketide synthase (PKS) reaction is involved in condensing acetate units onto the *N*-methyl-Δ^1^-pyrrolinium cation (**19**) leading to the second ring closure of TA biosynthesis [77,80,83]. Type III PKSs are a family of enzymes known to catalyze the iterative decarboxylative condensation of malonyl-CoA onto CoA-tethered substrates. Type III PKSs are promiscuous enzymes that have a broad tolerance for diverse substrates and are able to catalyze multiple reactions [84,85]. Type III PKSs that use cyclic nitrogen-containing substrates have been previously characterized for their roles in alkaloid production [86,87,88]. However, unlike these previous studies the predicted substrate in TA metabolism, *N*-methyl-Δ^1^-pyrrolinium cation (**19**) is charged and lacks a CoA thioester.

Spontaneous decarboxylation at the C2 position following ring closure can be prevented by the formation of the methyl ester. This would explain the presence of the carbomethoxy group found in cocaine (**1**) as well as other tropane alkaloids produced by plants found in the Erythroxylaceae. The resulting compound methylecgonone (**21**), contains a keto function at the C3 position and a carbomethoxy group at the C2 position [5]. Reduction at C3 is necessary for ester formation to occur.

**Scheme 3:**
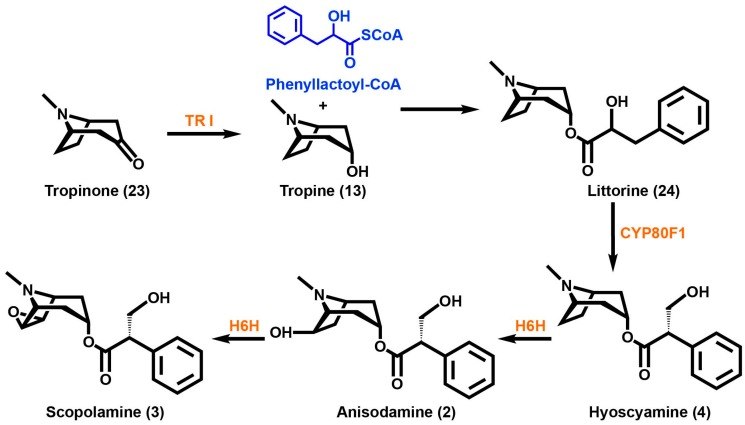
Tropinone is converted into tropine via TRI, tropinone reductase I. Tropine utilizes phenyllactoyl-CoA to yield littorine, which then uses a cytochrome p450 enzyme to form hyoscyamine. Scopolamine is epoxidized by the enzyme H6H, 6β-hydroxy hyoscyamine epoxidase, in a two-step process. Enzymes are highlighted in orange and substrates are highlighted in blue.

For the members of the Solanaceae, tropinone reductase (TR) enzymes catalyze the reduction of keto groups in the tropane ring. These enzymes are part of the short chain dehydrogenase/reductase (SDR) family that catalyzes NAD(P)(H)-dependent monomeric oxidoreductase reactions [89]. Its activity controls the metabolic flux towards TA biosynthesis downstream [90]. In solanaceous plants, two types of tropinone reductases exist, tropinone reductase I (TR I) and tropinone reductase II (TR II). They share a common tertiary “Rossman” fold structure, a conserved motif that consists of two pairs of α-helices and six parallel β-sheets, a catalytic active site with the motif YxxxK, and a dinucleotide cofactor-binding motif [91]. These enzymes share more than 50% of amino acid sequence similarity and are also assumed to have evolved from a common ancestor [90]. As little as five amino acid differences are needed to change the stereochemistry of the product [92]. TR I only converts the 3-keto function to a product that has a 3α-configuration (Scheme 3). This produces tropine (**13**) (3α-tropanol), which serves as a precursor for a wide range of esterified TAs [5]. On the contrary, TR II produces an alcohol solely with a 3β-configuration called pseudotropine (3β-tropanol). This is then converted to different nonesterified TAs called calystegines. A gene duplication event is attributed to these two different TRs in the Solanaceae family [93].

**Scheme 4:**
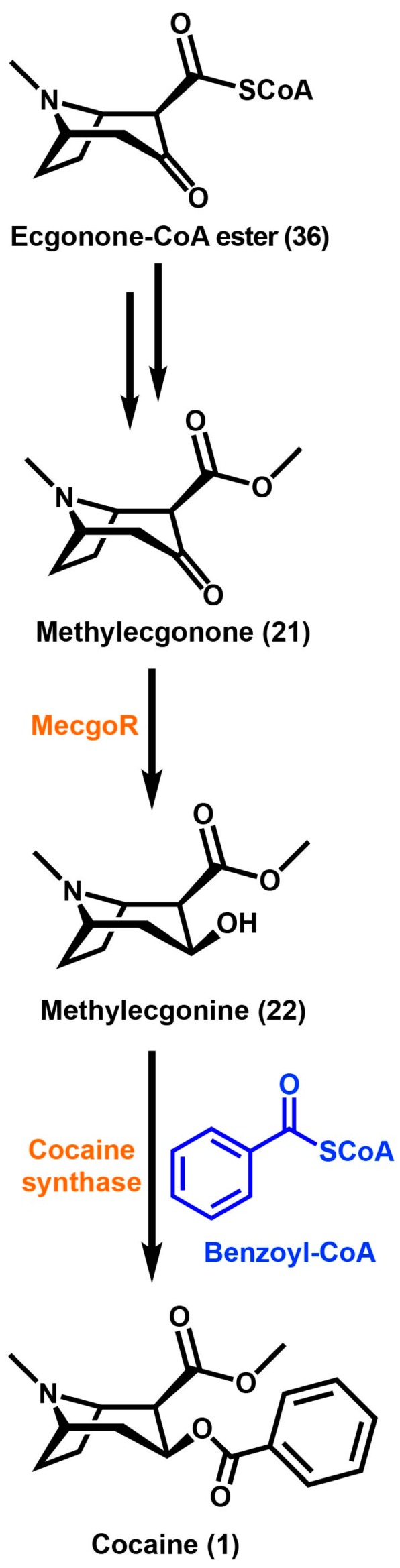
Ecgonone-CoA ester undergoes a multi-step process to yield methylecgonone, which is reduced by methylecgonne reductase, MecgoR to produce methylecgonine. Cocaine is then formed by the acylation of methylecgonine with benzoyl-CoA. Enzymes are highlighted in orange and substrates are highlighted in blue.

However in *E. coca*, a very different type of reductase enzyme was found for the reduction of the 3-keto function of methylecgonone (**21**) (Scheme 4). This is the first evidence of a polyphyletic origin for TA biosynthesis in plants. Methylecgonone reductase (MecgoR) was purified from crude protein extracts from coca leaves using classical biochemical techniques [5]. This enzyme was found to be very different than the TR enzymes that catalyzed the reduction reaction of the 3-keto function in solanaceous plants. MecgoR shares an overall identity of less than 10% at the amino acid level when compared to any TRs. MecgoR belongs to an aldo-keto reductase (AKR) superfamily of enzymes. AKRs that have been characterized so far share a common α/β-barrel motif, using either NADH or NADPH as a cofactor [94]. The MecgoR protein is localized in the palisade parenchyma of young developing leaves. This is in contrast to the localization of roots in TRs of solanaceous plants. This enzyme is also similar to other enzymes of alkaloid metabolism such as codeinone reductase and an enzyme of flavonoid biosynthesis, chalcone reductase. The stereospecific enzyme MecgoR catalyzes the conversion of methylecgonone (**21**) to the 3β-hydroxy-containing compound methylecgonine (**22**). MecgoR can also use tropinone (**23**) as a substrate but this only produces pseudotropine, which is consistent in *E. coca* with the presence of only 3β-hydroxy esters. Common TAs are esterified with aromatic or aliphatic acids, with the stereochemistry of the hydroxyl group dependent on the reductase used.

The benzoic ester of methylecgonine (**22**) is cocaine (**1**). Methylecgonine (**22**) is a molecule that has little physiological activity until it is converted into cocaine (**1**) [95]. There has been a prediction that an acyltransferase in *E. coca* utilizes benzoyl-CoA as the activated acid. This prediction was based on feeding studies using *trans*-[3-^13^C,^14^C]-cinnamic acid and the *N*-acetylcysteamine thioester of [3-^13^C,^14^C]-*trans*-cinnamic acid [96]. Methylecgonine (**22**) undergoes esterification with a benzoyl moiety that was predicted to utilize benzoyl-CoA as the activated acyl donor [97]. It was not determined if it arises from benzoyl-CoA or benzaldehyde, but the moiety was found to be derived from cinnamic acid [96,97]. Acylation reactions of secondary metabolites in plants are catalyzed by several acyltransferase families, however only the BAHD acyltransferase is known to utilize the activated acyl-CoA thioesters [98]. TAs are modified through the esterification of the hydroxyl function at the C3 position in the tropane ring. It has been established that in *E. coca*, the cocaine synthase reaction uses benzoyl-CoA and methylecgonine (**22**) as substrates. With the CoA-dependent nature of this enzyme along with the reported properties for the tigloyl-CoA:pseudotropine acyltransferase from *D. stramonium*, it was hypothesized that cocaine synthase is a member of the BAHD acyltransferase superfamily [99]. This superfamily of enzymes is well known to participate in secondary metabolite modification of esters and amides [98]. Cocaine synthase was found to be capable of producing both cocaine (**1**) via activated benzoyl-CoA thioester and cinnamoylcocaine via activated cinnamoyl-CoA thioesters [100]. It has been determined that the acylation of the 3β-hydroxyl function of methylecgonine (**22**), catalyzed by cocaine synthase, forms cocaine (**1**) and other TAs in *E. coca*. The accumulation and biosynthesis of TAs in *E. coca* occur within the same tissue. Cocaine synthase is found localized in the parenchyma and spongy mesophyll of the leaves. These tissues are both responsible for the biosynthesis and storage of TAs in this species. 4-coumaroylquinate has been reported to assist in the storage of cocaine (**1**) and cinnamoylcocaine in *E. coca* [101]. This is in contrast to TAs produced in the Solanaceae family where the core biosynthetic pathway is in the roots, while the metabolites accumulate in the aboveground portions of the plants [70].

The rearrangement of the hydroxyl group of the phenyllactic acid moiety of littorine (**24**) in TA side chain biosynthesis has drawn interest. The phenomenon occurs in both atropine and scopolamine (**3**) biosynthesis. A branched-chain residue, tropic acid, is formed from the linear chain phenyllactic acid. To better understand this process, feeding studies of radiolabeled compounds have tried to elucidate the mechanism of this reaction [102,103,104,105,106]. Feeding studies and quantum chemistry calculations by Sandala et al. have led to the hypothesis that a cytochrome p450 coupled with an alcohol dehydrogenase is involved in the conversion of the littorine (**24**) precursor into hyoscyamine (**4**) [107]. Li et al. were able to suppress cytochrome p450 CYP80F1 expression by using virus-induced gene silencing techniques that caused reduced levels of hyoscyamine (**4**) and promoted the accumulation of littorine (**24**) [108]. Using arylfluorinated analogues of (*R*)- and (*S*)-littorine, Nasomajai et al. were able to determine that the CYP80F1 catalyzed hydroxylation occurs via a benzylic carbocation intermediate [109]. Reversible 3′-acetoxylation of hyoscyamine (**4**) is thought to control the flux from hyoscyamine (**4**) to scopolamine (**3**) [110].

Hyoscyamine (**4**) is converted into the epoxide scopolamine (**3**) via hyoscyamine 6β-hydroxylase (H6H). This enzyme was shown to be a 2-oxoglutarate-dependent dioxygenase from purified *H. niger* [111,112]. Hydroxylation at the C6 position of hyoscyamine (**4**) followed by the epoxidation of anisodamine (**2**) (6β-hydroxy hyoscyamus) is catalyzed by H6H. The localization of this enzyme was also determined to be exclusively in the pericycle of roots [113]. Some solanaceous species contain acylations at the C6, C7 and C3 positions [110]. In a recent networking analysis study of the tropane biosynthetic pathway in *D. innoxia*, an enzyme activity was theorized in which acylation at the C3 position with a tiglic acid occurs using a C6 acylated tropane as the substrate [110]. By extension, Nguyen et al. have suggested that there are alternate C6-hydroxylating enzymes present with specificities different from H6H that could use tropinone (**23**) as a substrate instead of the reduced tropine (**13**) derivative. The same networking study revealed that high variability exists for the acylated tropanes which directly contributes to their chemical diversity [110]. Interestingly, some of the 3-hydroxyl acylating enzymes appear to not be stereospecific. Such enzymes could be useful for expanding the biocatalytic tools available to those interested in metabolic engineering or synthetic biology of tropane derivatives.

## 3. Granatane Alkaloid Biosynthesis

In this review, our definition of GAs includes piperideine derived compounds that incorporate a pelletierine (**5**) or *N*-methylpelletierine (**7**) core structure. There has been some confusion among scientists regarding the naming and configuration of pelletierine (**5**). Older reports sometimes use the name isopelletierine incorrectly to refer to the compound [*R*-1-(2-piperidyl) propan-2-one], which in reality corresponds to pelletierine (**5**). The name isopelletierine is the optically inactive racemate of pelletierine (**5**) [114]. The correct chemical name for the compound *N*-methylpelletierine (**7**) is 1-[(2*R*)-1-methyl-2piperdinyl]-2-propanone. Pseudopelletierine (**6**), also referred to as granatanone, contains a bicyclic core and can be referred to as [9-methyl-9-azabicyclo [1,3,3] nonan-3-one]. Additionally, the presence of anabasine (**8**), in *Nicotiana* species would by extension mean that selected members of the Solanaceae can be classified as granatane producing members. Furthermore, the solanaceous species *W. somifera* produces anabasine (**8**), which could also be classified as a GA. In addition, several *Sedum* species (family Crassulaceae) produce the compounds pelletierine (**5**), *N*-methylpelletierine (**7**), and pseudopelletierine (**6**). All of the species discussed above are members of the order Solanales and are more closely related to one another than they are to other granatane producing angiosperms (Figure 3). This may suggest that, at least within this order of angiosperms, the biosynthetic pathways leading to either tropanes or granatanes are commonly derived. The last time members of the Solanales shared a common ancestor with granatane producing lines in the Myrtales is approximately 120 million years ago. This is similar to the distance between the tropane producing Erythroxylaceae and Solanaceae.

The only research studies performed with the aim to demonstrate the biochemical precursors of GAs have been performed by feeding plants radiolabeled precursors. Table 1 provides a comprehensive summary of feeding studies performed in granatane-producing species as of this review. The labeled products were then subjected to analysis via chemical breakdown and modification. While these studies were informative, they can also be misleading when attempting to interpret them through the viewpoint of biochemical enzymes and mechanisms. The general consensus is that lysine (**25**) is the starting substrate for the entry into granatane biosynthesis. In several cases, different forms of labeled lysine were found incorporated into the core granatane structure [115,116,117,118,119,120].

Over the course of studies regarding granatane and piperideine alkaloid biosynthesis, there has been some controversy over the origin of the intermediates in the biosynthetic pathway. One of the main questions that has repeatedly been tested is whether or not cadaverine (**26**) is present in the pathway to form pelletierine (**5**) and other granatane derivatives. Several contradictory observations have been made depending on the type of label of the compound fed to plants as well as which species was used. The majority of the debate regarding the beginning intermediates arises when older radiolabel feeding studies in the *Sedum* species are used as a reference. These studies report the asymmetrical incorporation of the starting precursors into their respective alkaloids. However, other studies performed on pomegranate report that a symmetrical intermediate (such as cadaverine (**26**)) must exist [122]. Possible explanations for this problem could be that the members of the family Crassulaceae (e.g., *Sedum* species) do not utilize the same enzymatic steps as other granatane producing members such as those found in the family Lythraceae.

**Scheme 5:**
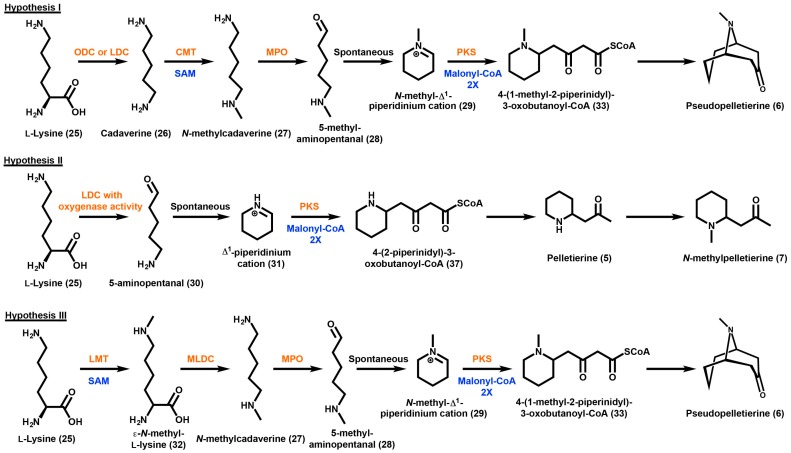
Three proposed hypothetical routes to the production of granatane alkaloids. Hypothesized enzymes are presented in orange and hypothetical cofactors are presented in blue. The abbreviated enzymes and cofactor are as follows: ODC, ornithine decarboxylase; LDC, lysine decarboxylase; CMT, cadaverine *N*-methyl transferase; MPO, methyl putrescine oxidase; PKS, polyketide synthase; LMT, lysine *N*-methyl transferase; MLDC, *N*-methyllysine decarboxylase; SAM, *S*-adenosyl-l-methionine.

The three hypothesized biosynthetic pathways for the production of GAs will be referred to as Hypothesis I, Hypothesis II, and Hypothesis III; their schematic representation is shown in Scheme 5. Hypothesis I is based on the observation that *P. granatum* plants fed [1.5-^14^C] and [1.5-^14^C ^3^H_2_] cadaverine yields incorporation of the labels into *N*-methylpelletierine (**7**) and pseudopelletierine (**6**) [121]. In this pathway, lysine (**25**) is first decarboxylated to form cadaverine (**26**) which would subsequently be methylated to form *N*-methylcadaverine (**27**) [119]. *N*-methylcadaverine (**27**) would then be oxidized to form 5-methylaminopentanal (**28**) promoting a spontaneous cyclization to form the *N*-methyl-∆^1^-piperidinium cation (**29**). This product would then ultimately form *N*-methylpelletierine (**7**) or be converted to the corresponding oxobutanoate by the decarboxylative condensation of two malonyl-CoA which would then cyclize to form pseudopelletierine (**6**). Feeding studies performed in *Sedum* species report that cadaverine (**26**) is not present as an intermediate, giving rise to Hypothesis II. By feeding [1-^14^C] cadaverine, Leistner et al. (1990) have predicted that there should be a lysine decarboxylase enzyme that also contains oxidase activity such that the only cadaverine (**26**) intermediate that exists is always enzyme bound [122]. Therefore, according to this hypothesis, lysine (**25**) is catalyzed in one enzyme or enzyme complex to produce 5-aminopentanal (**30**). The 5-aminopentanal intermediate (**30**) would cyclize to form a ∆^1^-piperidinium cation (**31**), which can then be methylated at the nitrogen atom to form the *N*-methyl-∆^1^-piperidinium cation (**29**) or remain unmethylated and ultimately go on to form pelletierine (**5**).

In all three hypotheses the origin of the *N*-methyl group is via labeled methionine [121]. The biochemical methyl donor for this incorporation would be S-adenosylmethionine (SAM). Hypothesis III also involves an early asymmetrical intermediate. In this hypothesis, the first biosynthetic step is the methylation of lysine (**25**) which occurs at the ε-*N* position producing ε*-N*-methyl-lysine (**32**). This compound would then be decarboxylated to form *N*-methylcadaverine (**27**) which would undergo oxidation to form 5-methylaminopentanal (**28**), promoting a spontaneous cyclization to form the *N*-methyl-∆^1^-piperidinium cation (**29**). As described in Hypothesis I, the *N*-methyl-∆^1^-piperidinium cation (**29**) would ultimately form *N*-methylpelletierine (**7**) and pseudopelletierine (**6**).

Radioactive acetate is incorporated into GAs regardless of which species is fed [116,117,118,119]. As is the case with tropane alkaloid biosynthesis, the enzymes involved in the extension and cyclization reactions of granatanes includes a putative polyketide synthase. Plants readily convert acetate into malonyl-CoA via the enzyme acetyl-CoA carboxylase [123]. A type III polyketide synthase utilizing 2 units of malonyl-CoA and the *N*-methyl-∆^1^-piperidinium cation (**29**) would produce 4-(1-methyl-2-piperinidyl)-3-oxobutanoyl-CoA (**33**). The prediction of the presence of a type III polyketide synthase in GA biosynthesis is supported by radiolabeling studies which fed ethyl (R,S)-[2,3-^13^C_2_,3-^14^C-]-4-(l-methyl-2-pyrrolidinyl)-3-oxobutanoate to *D. stramonium* plants [77,80,83]. The oxobutanoyl compound may then have several fates; its conversion to pseudopelletierine (**6**) by intermolecular interaction of the positively charged nitrogen and the carboxyl CoA, or its conversion to *N*-methylpelletierine (**7**) or pelletierine (**5**) by losing the carboxyl as CO_2_. Bicyclic GA producing species are similar to solanaceous plants producing tropanes, namely the loss of the carboxyl group due to a lack of methyl ester protection. As mentioned earlier in this review, the protection of the carboxyl group of oxobutanoate by methylation gives rise to the moiety responsible for the narcotic effects of cocaine (**1**), which would explain why GAs do not exhibit narcotic effects [18].

The enzymes responsible for carrying out the biochemical reactions described above are based on an extension of similar reactions carried out in tropane producing species. The decarboxylation of lysine (**25**) described in Hypothesis I would be performed by a *P. granatum* lysine decarboxylase (LDC). The presence of a lysine decarboxylase in the synthesis of anabasine (**8**) in *Nicotiana* species has been studied by the incorporation of [^15^N]-lysine into anabasine (**8**) [124]. The methylation of cadaverine (**26**) would be achieved with the help of a *P. granatum* cadaverine *N*-methyltransferase (CMT). CMTs have not been isolated and characterized in other species, but this enzyme is predicted to be related to putrescine *N*-methyltransferases (PMT) and spermidine synthases (SPDS). PMT cDNA sequences have been found in *Nicotiana* species by the sequencing of large genomic libraries [125,126]. The enzymes SPDS and PMT have been found to share substrate specificity, but PMT is dependent of the decarboxylated S-adenosylmethionine (dcSAM) as a co-substrate. The oxidation of *N*-methylcadaverine (**27**) in *P. granatum* and *Sedum* species could be performed with the aid of an enzyme similar to the methylputrescine oxygenase (MPO) present in the biosynthesis of nicotine in *N. tabacum*. While a copper dependent oxidase is used for tropane alkaloid biosynthesis, it is not possible to rule out alternative enzymes such as polyamine oxidases that require FAD as a cofactor [127]. The methylation of lysine (**25**) described in Hypothesis III would be performed by a lysine methyltransferase (LMT). The responsible enzyme may be related to the lysine methyltransferases ubiquitously present in eukaryotic primary metabolism for gene access regulation to chromatin [128].

In both granatane and tropane alkaloid producing species, dimerized versions of intermediates within their respective pathways have been found. For example, cuscohygrine is the dimerized form of hygrine (**20**) [129]. Originally it was believed that hygrine (**20**) was a true intermediate of the biosynthesis of TAs. However, it is most likely that cuscohygrine is a dimerized product of hygrine (**20**) which in turn is a breakdown product of 4-(1-methyl-2-pyrrolidinyl)-3-oxobutanoyl-CoA (**34**). If this compound is present as a free acid under physiological conditions a β-ketoester is formed, β-ketoesters very often spontaneously decarboxylate [130]. In the case of GA biosynthesis, anaferine (**9**) is the dimerization product of pelletierine (**5**). This also supports the presence of an oxobutanoate intermediate.

## 4. Metabolic Engineering

There has been an increasing interest in the biosynthesis of tropane alkaloids (TAs), especially to up-regulate the production of valued compounds, such as atropine and scopolamine (**3**). The World Health Organization (WHO) includes these important pharmaceutical compounds on their list of essential drugs [131]. In normal plant biosynthesis, the yields of the final compounds are in low quantities. Synthesizing TAs chemically in the lab has also been difficult and costly because of their stereochemical nature. Nocquet et al. attempted a total synthesis approach to produce the compound scopolamine (**3**), however their low yield of 16% does not make this method economically feasible [2]. A major problem for the commercial production of scopolamine (**3**) in hairy root cultures is achieving industrial level yields [132]. Researchers are now focused on metabolic engineering plants that produce these important compounds to increase final yields, or engineering microorganisms that will be able to produce the compounds from simple sugars or common precursors. A comprehensive table of the most recent metabolic engineering studies targeting specific genes can be seen on Table 2.

Due to its high demand in medicine, scopolamine (**3**) is the most popular choice for increasing yields via metabolic engineering. Past methods such as genetic breeding, polyploid breeding and radiation breeding have failed to yield a higher content of scopolamine (**3**) in *A. belladonna* [138]. Researchers are now focused on the genes encoding rate-limiting enzymes in TA biosynthesis that can be genetically modified *in planta*. A common focal point centers on what is considered the first and last rate-limiting enzymes in the TA pathway, putrescine *N*-methyltransferase (PMT) and hyoscyamine 6β-hydroxylase (H6H). The overexpression of only one *PMT* gene in transgenic hairy root cultures of *A. belladonna* did not change the total TA content [139]. If the same *PMT* gene in *D. metel* was overexpressed, the TA content was significantly increased by almost four times that of the control [140]. H6H has a high catalytic efficiency for converting hyoscyamine (**4**) to scopolamine (**3**) [141]. The overexpression of the *H6H* gene resulted in an increase in the biosynthesis of scopolamine (**3**) in the transformed TA producing plant, *A. belladonna*. Another successful use of *H6H* was in transgenic *Hyoscyamus muticus* hairy root cultures, where scopolamine (**3**) levels increased to over 100 times that of the controls [142]. Furthermore, overexpression of *H6H* in transgenic *A. belladonna* plants resulted in the leaf and stem alkaloid contents to be exclusively scopolamine (**3**) [141].

Metabolic engineering endeavors are becoming more complex and are moving away from only modifying one gene at a time. When both *PMT* and *H6H* were overexpressed simultaneously in transgenic *H. niger* root cultures, scopolamine (**3**) biosynthesis increased to levels over nine times more than the wild type [143]. In an important experiment testing whether metabolic engineering can occur between genes isolated from different species, overexpression of *NtPMT* and *HnH6H* in *A. belladonna* significantly increased scopolamine (**3**) content of secondary roots when compared to wild-type plants [11]. More studies are needed to understand flux through the tropane biosynthetic pathway in order to increase the overproduction of alkaloids.

*D. metel* produces important medicinal tropanes and is used by researchers because of its tractable hairy root culture system. *Agrobacterium rhizogenes* can transform plant roots to hairy roots by utilizing the Ri T-DNA plasmid it carries. Hairy roots that have been induced by *A. rhizogenes* have high growth rates, are genetically stable, and produce copious amounts of lateral roots [144]. Increased TA biosynthesis correlated with an increase in root biomass. Biotic elicitors, such as yeast extract, bacteria, fungi and viruses, as well as abiotic elicitors, such as metal ions or inorganic components, are used and studied to increase the productivity of hairy roots. These elicitors can trigger different defense responses and phytoalexins in plants as well as improve the release of metabolites into the medium [10]. Shakeran et al. focused on using *Staphylococcus aureus* and *Bacillus cereus* as biotic elicitors and silver nitrate and nanosilver as abiotic elicitors on the hairy root cultures of *D. metel* to see their effects on biomass and atropine production. When live bacteria are present in transformed root cultures, there is a considerable influence on secondary metabolite accumulation [145]. Contrary to this, atropine content in the hairy roots of *D. metel* infected by *B. cereus* and *S. aureus* was reduced more than half when compared to the control. The authors hypothesize that this may be a cause of atropine secretion into the culture medium that was then converted into scopolamine (**3**) [10]. However, scopolamine (**3**) was not analyzed in the spent media. The living bacteria can cause various influences on roots, affecting enzymes in the TA pathway to produce alkaloids in *D. metel* roots [145]. Although atropine accumulation decreased with these biotic elicitors, the biomass of the roots slightly increased approximately 15% when compared to the control [10].

Other attempts to engineer higher TA contents in plants include those that use abiotic elicitors. A summary of recent metabolic engineering studies using elicitors can be seen on Table 3. Silver nitrate can increase anisodamine (**2**) content in *Anisodus acutangulus* hairy root cultures [146]. In addition, calcium and nitrate can increase hyoscyamine (**4**) content in *D. stramonium* hairy root cultures [147]. Silver nitrate can inhibit the activation of ethylene and in doing so, promotes polyamine synthesis [148,149]. As a consequence, the overall effect of silver nitrate treatment can be seen in the increase of root biomass [148,149,150]. Furthermore, silver nitrate has been demonstrated to elicit the production of phytoalexins which in turn increases TA levels in the root [151]. 

Shakeran et al. (2015) used silver nitrate as an abiotic elicitor in *D. metel* and observed an increase in the transformed hairy root biomass of approximately 16%. However, only half of the expected atropine accumulation was observed in these treatments. Although secretion of atropine was not measured in this study, subsequent reports corroborated this hypothesis by finding a three-fold increase in alkaloids in the spent culture media following silver nitrate treatment [152]. Still, further attempts at increasing alkaloid levels include the treatment using nanosilver particles. These differ from silver nitrate in their physicochemical properties [153,154]. Nanosilver particles adhere strongly to plant tissues and cause an increase in the activation of enzymes involved in secondary metabolite production. This treatment was used successfully in *Artemisia annua* and *D. metel* hairy roots increasing tropanes by at least 2.4 fold [10]. Initial polyamine substrates such as putrescine (**14**) must be present in abundance during TA production for a high yield of the final alkaloid. Currently there are only a few studies attempting to engineer the TA pathway in microorganisms. Qian et al. engineered a strain of *Escherichia coli* capable of efficiently producing putrescine (**14**) [134]. However, it was first necessary to reduce the flux of polyamine precursors through a competing pathway. Metabolic pathways for putrescine (**14**) degradation, uptake and utilization were also deleted. Stress to cells by the overproduction of putrescine (**14**) was handled by the deletion of RpoS, a stress responsive RNA polymerase sigma factor. To increase the conversion of ornithine (**10**) to putrescine (**14**), overexpression of ornithine biosynthetic enzymes and ornithine decarboxylase (ODC) was also necessary. The final metabolically engineered *E. coli* strain produced 1.68 g/L of putrescine (**14**) and high cell density cultures (HCDCs) produced 24.2 g/L of putrescine (**14**). This would be the first step for engineering alkaloid biosynthesis that relies on putrescine (**14**) in microorganisms. In a follow-up study, Qian et al. (2011) performed similar manipulations to produce a strain of *E. coli* capable of producing the polyamine cadaverine (**26**). Introduction of an l-lysine decarboxylase in addition to overexpressing *dapA*, the gene encoding the enzyme dihydrodipicolinate synthase successfully resulted in the new strain producing as much as 9.61 g/L cadaverine (**26**) from renewable resources [155]. If metabolic engineers in the future wish to engineer these pathways in other organisms, such as bacteria or yeast, it will be necessary to up-regulate the beginning precursor pathways such that pools of these primary metabolites do not get depleted. Depletion of these essential metabolites would result in the death of the organism during the biosynthesis of these compounds.

Using plant in vitro cell culture or tissue culture is an important tool when studying the regulation and biosynthesis of secondary metabolites. Large quantities of plant material can be produced under controlled and sterile conditions. In tissue cultures of solanaceous plant species, using elicitors mimicking stress hormones can increase important secondary metabolite production [158]. Recently, cell cultures of *E. coca* in the Erythroxylaceae were used to study TA biosynthesis [157]. Various culture media were tested on their ability to support callus formation as well as cocaine (**1**) production. The jasmonic acid-isoleucine (JA-Ile) analogue coronalon and salicylic acid (SA) were also used as elicitors to observed their effects on calli metabolism. All three culture media growing calli accumulated cocaine (**1**). The medium used to grow calli also significantly affected natural product metabolism. The only treatments that yielded higher amounts of cocaine (**1**) were dependent upon culture media, not upon elicitor treatment. For example, Anderson rhododendron medium (ARM) produced cocaine (**1**) an order of a magnitude greater than both Gamborg B5 (GB5) and modified Murashige-Tucker medium (MMT), but lower levels of hydroxycinnamate-quinate esters such as chlorogenic acid (CGA) were detected. Interestingly the elicitors coronalon and salicylic acid did not yield any increase in TA production suggesting that TAs, at least in *E. coca*, may not be regulated by common plant defense hormones.

## 5. Conclusions

Recent advances in genomics, transcriptomic and metabolomic technologies are poised to illuminate the biosynthetic foundations of TAs and GAs. Future research on the biosynthesis of TAs and GAs will affect multiple fields of research. First, enzymes involved in TA and GA biosynthesis will expand our fundamental knowledge of chemistry and enzymology. Second, elucidation of the genes and enzymes underlying TA and GA biosynthesis will expand the molecular tools available to synthetic biologists. With these additional tools, scientists can look to not only produce TAs and GAs in heterologous hosts but also to engineer novel molecules [159]. Lastly, future discoveries of the function and structure of genes and enzymes in TA and GA biosynthesis will strengthen our understanding of the evolution of plant metabolism. Plant metabolism, especially specialized metabolites such as TAs and GAs, evolved in response to dynamic environments. Detailed knowledge of the catalytic properties of TA and GA biosynthetic enzymes as well as their biophysical properties are critical to our knowledge of the evolution of biochemical activities and the chemical diversity of these metabolic pathways. This fundamental knowledge will be useful in predicting and engineering plants to withstand ongoing changes in the environment.

## Figures and Tables

**Table 1:** Summary of radiolabel feeding studies performed on granatane-producing species.

Species Studied	Radiolabeled Compound	Where Label Was Found	Reference
*Punica granatum*	1-^14^C-acetate	*N*-methyl isopelletierine	[119]
*Punica granatum*	2-^14^C-lysine	*N*-methyl isopelletierine	[119]
*Withania somnifera*	2-^14^C-lysine	anaferine	[119]
*Punica granatum*	[1-^14^C] acetate	Isopelletierine, *N*-methylisopelletierine, and pseudopelletierine	[118]
*Withania somnifera*	[1-^14^C] acetate	anaferine	[118]
*Punica granatum*	dl-[2-^14^C] lysine	*N*-methylisopelletierine asymmetrically	[118]
*Withania somnifera*	dl-[2-^14^C] lysine	Anaferine asymmetrically	[118]
*Punica granatum*	[N-*methyl-^14^*C, 8^14^C] methylisopelletierine	pseudopelletierine	[118]
*Withania somnifera*	[8-^14^C] isopelletierine	anaferine	[118]
*Punica granatum*	[1,5^15^C] cadaverine and [^3^H] cadaverine	*N*-methylisopelletierine and pseudopelletierine nonrandomly	[121]
*Punica granatum*	[^14^C] methionine	*N*-methylisopelletierine and pseudopelletierine	[121]
*Sedum sarmentosum*	Sodium [1,2,3,4-^13^C_4_] acetate and sodium [1,2-^13^C_2_] acetate	*N*-methylpelletierine	[116]
*Sedum acre* and *Sedum sarmentosum*	dl-[6-^14^C] lysine, l-[4,5-^8^H_2_] lysine, and d-[6-^14^C] lysine	Only l enantiomer of lysine was incorporated into *N*-methylpelletierine.	[115]
*Nicotiana glauca*	dl-[6-^14^C] lysine, l-[4,5-^8^H_2_] lysine, and d-[6-^14^C] lysine	Only l enantiomer of lysine was incorporated into anabasine.	[115]
*Sedum sarmentosum*	[6-^14^C] lysine	*N*-methylisopelletierine	[120]
*Sedum sarmentosum*	6-^14^C-dl-lysine	*N*-methylpelletierine asymmetrically	[117]
*Sedum sarmentosum*	4,5-^3^H_2_,6-^14^C-dl-lysine	N-methylpelletierine	[117]

**Table 2:** A summary of recent metabolic engineering studies targeting specific genes.

Species	Target Compound	Gene/Genes Modified	Effect	Reference
*Atropa belladonna*	Scopolamine	*NtPMT* & *HnH6H*	Increased scopolamine content	[11]
*Atropa belladonna*	Hyoscyamine & scopolamine	*rolC*, *pmt*, & *h6h*	Increased hyoscyamine content & increased scopolamine content	[133]
*Escherichia coli*	Putrescine	Multiple	Increased putrescine production	[134]
*Brugmansia condida*	Polyamines (putrescine)	*rolC*	Polyamine accumulation, improve hairy root growth	[135]
*Atropa belladonna*	Pseudotropine/Tropine	*tr-1*/*tr-2*	Higher enzyme activity & increase in pseudotropine/tropine	[136]
*Anisodus acutangulus*	TAs	*pmt* & *tr-1*	Increased TA levels with hyoscyamine being major alkaloid	[137]

**Table 3:** A summary of recent metabolic engineering studies using elicitors.

Species	Target Compound	Elicitor	Effect	Reference
*Anisodus luridus*	Scopolamine	Acetylsalicylic acid (ASA)	Increased scopolamine content	[156]
*Anisodus luridus*	Scopolamine	Ultraviolet ray-B (UV-B)	Increased scopolamine content	[156]
*Datura metel*	Atropine	*Staphylococcus aureus*	Decreased atropine content, increased root biomass	[10]
*Datura metel*	Atropine	*Bacillus cereus*	Decreased atropine content, increased root biomass	[10]
*Datura metel*	Atropine	Silver nitrate	Decreased atropine content, increased root biomass	[10]
*Datura metel*	Atropine	Nanosilver	Increased atropine content, increased root biomass	[10]
*Datura innoxia*	Hyoscyamine	*Agrobacterium rhizogenes*	Increased hyoscyamine content, increased root biomass	[110]
*Erythroxylum coca*	Cocaine	Anderson rhododendron medium (ARM)	Increased cocaine content in calli	[157]
*Erythroxylum coca*	Chlorogenic acid (CGA)	Salicylic acid	Decreased CGA content	[157]
